# Pathological features of 11,337 patients with primary ductal carcinoma in situ (DCIS) and subsequent events: results from the UK Sloane Project

**DOI:** 10.1038/s41416-020-01152-5

**Published:** 2020-11-17

**Authors:** Abeer M. Shaaban, Bridget Hilton, Karen Clements, Elena Provenzano, Shan Cheung, Matthew G. Wallis, Elinor Sawyer, Jeremy S. Thomas, Andrew M. Hanby, Sarah E. Pinder, Alastair M. Thompson, on behalf of the Sloane Project Steering Committee

**Affiliations:** 1 Queen Elizabeth Hospital Birmingham and University of Birmingham, Birmingham, UK; 2 Screening Quality Assurance Service, Public Health England, Birmingham, UK; 3 Addenbrookes Hospital, Cambridge, UK; 4 Cambridge Breast Unit, and NIHR Cambridge Biomedical Research Centre, Cambridge University Hospitals NHS Trust, Cambridge, UK; 5 School of Cancer & Pharmaceutical Sciences, King’s College London and Guy’s and St Thomas’ Hospitals NHS Foundation Trust, London, UK; 6 Western General Hospital, Edinburgh, UK; 7 Leeds Institute of Medical Research at St. James’s, St James’s University Hospital, Leeds, UK; 8 Baylor College of Medicine, Houston, TX USA

**Keywords:** Breast cancer, Cancer screening

## Abstract

**Background:**

The Sloane audit compares screen-detected ductal carcinoma in situ (DCIS) pathology with subsequent management and outcomes.

**Methods:**

This was a national, prospective cohort study of DCIS diagnosed during 2003–2012.

**Results:**

Among 11,337 patients, 7204 (64%) had high-grade DCIS. Over time, the proportion of high-grade disease increased (from 60 to 65%), low-grade DCIS decreased (from 10 to 6%) and mean size increased (from 21.4 to 24.1 mm). Mastectomy was more common for high-grade (36%) than for low-grade DCIS (15%). Few (6%) patients treated with breast-conserving surgery (BCS) had a surgical margin <1 mm. Of the 9191 women diagnosed in England (median follow-up 9.4 years), 7% developed DCIS or invasive malignancy in the ipsilateral and 5% in the contralateral breast. The commonest ipsilateral event was invasive carcinoma (*n* = 413), median time 62 months, followed by DCIS (*n* = 225), at median 37 months. Radiotherapy (RT) was most protective against recurrence for high-grade DCIS (3.2% for high-grade DCIS with RT compared to 6.9% without, compared with 2.3 and 3.0%, respectively, for low/intermediate-grade DCIS). Ipsilateral DCIS events lessened after 5 years, while the risk of ipsilateral invasive cancer remained consistent to beyond 10 years.

**Conclusion:**

DCIS pathology informs patient management and highlights the need for prolonged follow-up of screen-detected DCIS.

## Background

Ductal carcinoma in situ (DCIS) is a heterogeneous disease that has increasingly been diagnosed in the context of mammographic screening. The natural history, optimal management and follow-up for DCIS remain controversial.^1^ The United Kingdom National Health Service Breast Screening Programme (NHS BSP) presently invites women aged 50–70 years to attend for 2 view mammography every 3 years. The Sloane Project, established in memory of the breast pathologist Professor John Sloane, is a prospective cohort study that examines the clinical, radiological and pathological features, patterns of care and outcomes for women with non-invasive neoplasia detected within the NHS BSP. Eighty-two of 94 (87%) UK NHS Breast Screening Units have submitted data yielding detailed information on one-third of all DCIS diagnosed via the NHS BSP during the time period.^2^

The Sloane Project provides a unique opportunity to explore unanswered questions regarding screen-detected DCIS. Despite previous randomised clinical trials^3,4^ and large numbers of single-centre studies on biomarkers,^5–8^ the optimal management and follow-up of patients with DCIS remains controversial. The issue of over-diagnosis/over-treatment of DCIS has been highlighted and ongoing clinical trials across the world are attempting to evaluate active surveillance strategies as an alternative to surgical excision for low-risk DCIS.^9–12^ Large, well-characterised, prospective, multicentre series of DCIS represent an invaluable resource providing real-world information. The consistency of reporting among histopathologists and the distribution of reported parameters, such as DCIS grade and size, the presence of comedo necrosis and microinvasion, are relevant for patient management and clinical trial entry and trends in the reporting of these over time have largely been unexplored. There is no robust data in the current literature on the incidence and patterns of atypical lesions associated with DCIS, the histological identification of which may also alter patient management. We have therefore analysed, in detail, the pathological features of a large prospective cohort of well-characterised screen-detected DCIS patients and associated atypical epithelial lesions within the Sloane Project, assessed changes in pathological features over time (2003–2012) and the development of subsequent ipsilateral, contralateral and distant metastasis events.

## Methods

Contributing Screening Units completed radiology, pathology, surgery and radiotherapy proformas prospectively.^13^ This included demographic, diagnostic, treatment and vital status data. A pathology protocol, based on the UK Royal College of Pathologists/BSP guidelines,^14^ advises on the handling and reporting of specimens to mandatory national standards and contains definitions and guidance for the diagnosis of DCIS, microinvasion as well as cytonuclear grading, comedo necrosis and the assessment and reporting of excision margins. Oestrogen receptor, progesterone receptor and HER2 expression are not routinely evaluated on DCIS in the UK. Here we present an analysis of the pathological features of DCIS and of subsequent events, if present, in 11,337 women diagnosed between 2003 and 2012 via breast screening. Subsequent events, including ipsilateral and contralateral DCIS and/or invasive disease and distant recurrence that developed ≥6 months following the primary DCIS diagnosis, were collected up to December 2016.^13^

To ensure robustness of information, including patient outcome, data searching and cross-checking across different databases was conducted to ascertain recurrences. This was performed by matching patient’s date of birth and NHS number to data collected from Breast Screening Units and other routinely collected sources of information, including Hospitals Episode Statistics, Cancer Waiting Times, the English Cancer Analysis System, National Cancer Registration and Analysis System and the English National Radiotherapy data sets.

### Statistical analysis

Stata was used for statistical analyses. For continuous variables, the percentage was calculated as a proportion of the total number. Some variables, such as size and margin status, were re-coded into categorical groups for further analysis. Where data were not normally distributed, Spearman’s rank correlation was employed. Logistic regression was used to calculate the odds ratio (OR) for the proportion of cases with high-grade DCIS and the proportion with microinvasion, with year as a factor and 2003/04 as baseline. Survival was defined as the time between initial DCIS diagnosis and diagnosis of recurrent breast cancer or metastases. Patients with no recurrence were censored at date of death/date last seen. All tests were two sided. A probability value of ≤0.05 was considered significant.

## Results

A summary of the DCIS characteristics of the whole cohort stratified by treatment type is presented in Table 1.

**Table 1:** DCIS characteristics of the whole cohort stratified by treatment groups.

	BCS only		BCS+RT		BCS (RT unknown)		Mx		Total	
**DCIS grade**										
Low	693	23.3%	114	2.5%	38	9.5%	151	4.5%	996	8.8%
Intermediate	1371	46.2%	979	21.4%	118	29.6%	633	18.8%	3101	27.4%
High	892	30.0%	3472	76.0%	239	60.1%	2591	76.7%	7194	63.6%
Unknown	13	0.4%	2	0.0%	3	0.8%	1	0.0%	19	0.2%
**DCIS architecture**										
Solid	1356	45.7%	3098	67.8%	240	60.3%	2183	64.7%	6877	60.8%
Cribriform	1709	57.6%	2173	47.6%	182	45.7%	1721	51.0%	5785	51.1%
Micropapillary	474	16.0%	554	12.1%	52	13.1%	774	22.9%	1854	16.4%
Flat	133	4.5%	189	4.1%	20	5.0%	261	7.7%	603	5.3%
Papillary	164	5.5%	168	3.7%	22	5.5%	153	4.5%	507	4.5%
Apocrine	65	2.2%	89	1.9%	3	0.8%	93	2.8%	250	2.2%
Other	23	0.8%	32	0.7%	3	0.8%	34	1.0%	92	0.8%
Unknown	266	9.0%	393	8.6%	46	11.6%	267	7.9%	972	8.6%
**Size of DCIS**										
<10 mm	1553	52.3%	1025	22.4%	103	25.9%	198	5.9%	2879	25.5%
10–20 mm	895	30.1%	1675	36.7%	150	37.7%	466	13.8%	3186	28.2%
20–30 mm	276	9.3%	1078	23.6%	80	20.1%	545	16.1%	1979	17.5%
30–4 mm	91	3.1%	443	9.7%	28	7.0%	509	15.1%	1071	9.5%
>40 mm	59	2.0%	311	6.8%	27	6.8%	1592	47.2%	1989	17.6%
No residual DCIS^a^	68	2.3%	19	0.4%	6	1.5%	7	0.2%	100	0.9%
Unknown	27	0.9%	16	0.4%	4	1.0%	59	1.7%	106	0.9%
**Comedo necrosis**										
Present	1097	36.9%	3211	70.3%	215	54.0%	2369	70.2%	6892	60.9%
Absent	1552	52.3%	984	21.5%	105	26.4%	711	21.1%	3352	29.6%
Unknown	320	10.8%	372	8.1%	78	19.6%	296	8.8%	1066	9.4%
**Associated epithelial atypia (ADH or lobular neoplasia)**										
Yes	500	16.8%	448	9.8%	40	10.1%	312	9.2%	1300	11.5%
No	2469	83.2%	4119	90.2%	358	89.9%	3064	90.8%	10,010	88.5%
**Microinvasion**										
Present	82	2.8%	325	7.1%	19	4.8%	313	9.3%	739	6.5%
Absent	2823	95.1%	4178	91.5%	344	86.4%	3018	89.4%	10,363	91.6%
Unknown	64	2.2%	64	1.4%	35	8.8%	45	1.3%	208	1.8%
**Margin width**										
<2 mm	375	13%	644	14%	65	16%		0%	1084	10%
≥2 mm	2410	81%	3719	81%	259	65%		0%	6388	56%
Unknown	184	6%	204	4%	74	19%		0%	462	4%
** Total number**	2969	100%	4567	100%	398	100%	3376	100%	11,310	100%

^a^No residual DCIS in the surgical excision. Lesion was removed by previous core/vacuum-assisted biopsy.

### Size of DCIS

The majority of DCIS lesions submitted to the Sloane Project over the period 2003–2012 were under 20 mm in extent (*n* = 6067, 54%) although 18% (1989) were >40 mm in size. There was a statistically significant increase in histological DCIS size, from a mean of 21.4 mm in 2003–2004 to 24.1 mm in 2011–2012 (Spearman rank correlation test, *p* < 0.001). This was largely due to an increase in the proportion of lesions reported as measuring 30 mm or more on histology and particularly an increase in the proportion of lesions measuring ≥40 mm. As might be expected, increasing lesion size was associated with a greater likelihood of patients undergoing mastectomy, with 80% of patients with DCIS >40 mm treated by mastectomy, compared to 19% of those with DCIS <40 mm in size.

### Grade of DCIS

Of the 11,337 women with primary DCIS, 7204 (64%) had high cytonuclear grade disease, 27% (3107) were of intermediate grade and 9% were of low grade; 27 (0.2%) were of unknown grade. Of note, an increase in the proportion of high-grade DCIS was noted from 60% of the total in 2004 to 65% in 2012 (60, 61, 62, 64, 62, 67, 64, 67 and 65% in consecutive years), coupled with a decrease in the proportion of low-grade DCIS, from 10 to 6% from 2003/04 to 2011/12. Logistic regression analysis comparing the years of diagnosis from 2003/04 to 2011/12 demonstrated that these trends were statistically significant (*p* = 0.011 for the increase in the proportion of high-grade DCIS and *p* = 0.001 for the decrease in the proportion of low-grade DCIS). Breast-conserving surgery (BCS) was the preferred mode of surgical treatment across all DCIS grades, but mastectomy was more common in patients with high-grade DCIS (36%) compared to those with low-grade DCIS (15%) (Chi^2^ 170.9, df = 1, *p* < 0.0001).

### Architecture of DCIS

Solid DCIS was the predominant architectural pattern, seen in 61% of cases; 72% of high-grade DCIS was of solid pattern compared with 49% of intermediate-grade and 21% of low-grade disease. The second commonest architecture was cribriform (51% of cases), accounting for 44% of high-grade DCIS and 63 and 68% of intermediate- and low-grade lesions, respectively. Micropapillary architecture was seen in 16% of all cases, more commonly of low or intermediate grade (27 and 18%, respectively) compared to high-grade DCIS (14%). A papillary architecture was seen in 4% of DCIS cases, less often of high (3%) than low- or intermediate-grade disease (7 and 8%, respectively). Flat architecture of DCIS was seen in 5% of all patients but, intriguingly, was reported in 4% each of low- and intermediate-grade lesions. Current international guidelines classify non-high-grade flat epithelial proliferations as flat epithelial atypia (FEA) rather than DCIS, although historically it was categorised as the monomorphic variant of clinging carcinoma, a term no longer recommended by the World Health Organisation (WHO) Classification of Tumours Editorial Board.^15^

The architectural pattern of DCIS was associated with the surgical management: patients with flat and micropapillary DCIS had the highest mastectomy rates (43 and 42%, respectively), with both these patterns exhibiting larger histological size (31 and 30% of flat and micropapillary DCIS, respectively measured ≥40 mm).

### Comedo necrosis

Comedo necrosis was reported in 61% of cases overall, most commonly in high-grade DCIS (78%). It was, however, seen in 39% of intermediate-grade DCIS and 6% of low-grade lesions. The percentage of DCIS reported as showing comedo necrosis varied considerably between Units; even excluding centres that contributed <20 DCIS cases, the reported incidence of comedo necrosis ranged from 22 to 84%.

### Margin clearance

Only 3% (233) of patients with BCS had radial margin clearance described as 0 mm (i.e. involved), with another 3% having disease <1 mm from the margin. The most common margin width reported was ≥10 mm (35%, 2814 patients) with 8% (614), 20% (1559) and 25% (2015) of women undergoing breast conservation having margin widths of 1–1.9, 2–4.9 and 5–9.9 mm, respectively (Table 2). In 2%, no margin width was described but the disease was classified as ‘clear’ and in 4% of cases the margin width was unknown. Thus some 14% of women had margins less than the 2 mm currently considered desirable in the UK and US. Of note, there was no significant difference in margin width in patients with DCIS reported to the Sloane Project between 2003 and 2012; there was no increase in those patients with <1 mm margin or conversely >5 mm margin widths over this time period.

**Table 2:** Minimum margin size recorded for patients who underwent breast-conserving surgery (BCS).

Margin size (mm)	BCS	Percent
0	233	3%
<1	237	3%
1–1.9	614	8%
2–4.9	1559	20%
5–9.9	2015	25%
≥10	2814	35%
Clear	122	2%
Unknown	340	4%
Total	7934	100%

### Microinvasion

Microinvasion was reported as present in 738 women (7%). This was more frequently seen in association with high-grade DCIS (8%) but was present in 4% of intermediate-grade and in 1% of low-grade DCIS. The frequency of microinvasion reported decreased from 9% in 2003/2004 to 5% in 2011/2012 and logistic regression confirmed a significant reduction in the presence of microinvasion by year (comparing 2003/04 to 2011/12; *p* < 0.001). Nevertheless, for the overall period, there was very wide variation in the percentage of microinvasion reported across the submitting UK pathology departments (range 0–29%).

### Associated atypia

Atypical ductal hyperplasia (ADH) was reported in association with DCIS (with no additional atypia such as lobular neoplasia or FEA) in 611 patients; this was less frequently seen in association with high-grade DCIS (3%) than with intermediate-grade (7%) or low-grade disease (14%) (1 unknown) (Table 3). In a further 111 patients, both ADH and lobular in situ neoplasia were present along with the DCIS, again more commonly in low-grade DCIS (4%) than in intermediate- (1%) or high-grade disease (<1%) (Table 3).

**Table 3:** Associated lesions by DCIS grade.

Associated lesion	DCIS grade									
	High	Percent	Intermediate	Percent	Low	Percent	Unknown		All patients	
None	6628	92%	2647	85%	752	75%	21	78%	10,048	89%
ADH alone	238	3%	228	7%	144	14%	1	4%	611	5%
LISN alone	294	4%	190	6%	64	6%	3	11%	551	5%
ADH and LISN	32	0%	40	1%	38	4%	1	4%	111	1%
PLCIS	12	0%	2	0%	1	0%	1	4%	16	0%
Total	7204	100%	3107	100%	999	100%	27	100%	11,337	100%

*ADH* atypical ductal hyperplasia, *LISN* lobular in situ neoplasia, *PLCIS* pleomorphic lobular carcinoma in situ.

Of the women with lobular neoplasia as the only additional atypical lesion (*n* = 294), 4% was recorded in patients with high-grade DCIS, 6% with intermediate and 6% in those with low-grade DCIS (in 3 cases, grade was not known). Pleomorphic lobular carcinoma in situ was uncommonly reported in the Sloane Project in patients with DCIS (16 in total); 12 of these women (75%) had high-grade DCIS (but this represented <1% of all high-grade DCIS lesions).

### Subsequent first events

Subsequent events are defined as the first development of in situ or invasive breast carcinoma in the ipsilateral or contralateral breast, or distant metastasis, >6 months following a diagnosis of DCIS. At the time of writing, up-to-date information was available for patients from England only (*n* = 9191), and consequently, the analysis has been limited to English patients and their first episode of recurrent disease.

Of these 9191 women, 1098 (12%) re-presented with DCIS or invasive malignancy in the ipsilateral (7%) or contralateral breast (5%) and 46 (0.5%) patients developed distant recurrence, at a median of 9.2 years (range 0.4–14.5) follow-up. Ipsilateral invasive disease (*n* = 413) was more common than ipsilateral DCIS (*n* = 225). A further 20 patients had an unspecified ipsilateral recurrence (not known if DCIS or invasive). Contralateral events were also more commonly invasive (*n* = 325) than in situ (*n* = 94). Detailed information on three recurrences was not available (Table 4).

**Table 4:** Patterns of breast and distant recurrences following high-, intermediate- and low-grade DCIS.

Primary lesion	Ipsilateral		Contralateral		Unknown side		Distant		Total	Unknown status
	*n*	Percent	*n*	Percent	*n*	Percent	*n*	Percent		
High-grade DCIS	377	6.6	249	4.3	3	0.05	35	0.4	5753	147
Intermediate-grade DCIS	210	8.2	142	5.6	0	0	8	0.27	2550	63
Low-grade DCIS	73	8.4	39	5.6	1	0.1	3	0.23	868	21
Unknown grade DCIS	3	0%	1	0	0	0	0	0	20	1
Total	663	100%	431	100%	4	100%	46	100%	9191	232

Overall, 377/5753 (6.6%) patients with high-grade DCIS had an ipsilateral recurrence compared with 210/2550 (8.2%) with intermediate-grade DCIS and 73/868 (8.4%) with low-grade disease. When only patients who underwent BCS are considered, the proportions with any ipsilateral recurrence were more comparable, amounting to 9.2, 9.7 and 9.8% of high-, intermediate- and low-grade DCIS, respectively. Invasive ipsilateral recurrence occurred overall after 3.7% of high-grade lesions, 5.7% of intermediate and 5.6% of those with low-grade DCIS; for patients undergoing breast conservation, the invasive recurrence frequencies were 4.9%, 6.7% and 6.7%, respectively.

There was a significant relationship between the grade of primary DCIS and that of the subsequent DCIS (*p* < 0.001, Table 5), whereas there was no statistically significant association between the grades of DCIS and subsequent invasive disease (*p* = 0.08, Table 5). Where data were available, subsequent invasive carcinoma was predominantly of grade 2 in the ipsilateral (46%) and the contralateral breast (52%), irrespective of the original DCIS grade. Following a diagnosis of high-grade DCIS, 37% of ipsilateral or 27% of contralateral invasive carcinomas were grade 3. Low-grade DCIS was followed by ipsilateral or contralateral invasive grade 3 carcinoma in 12 and 29% of cases, respectively.

**Table 5:** Ipsilateral DCIS recurrence and subsequent invasive carcinoma by primary DCIS disease.

Primary lesion	Low		Intermediate		High		Unknown		Total	*p* value
Recurrent DCIS grade										
High-grade DCIS	1	1%	16	11%	109	73%	24	16%	150	<0.001
Intermediate-grade DCIS	2	3%	20	34%	22	37%	15	25%	59	
Low-grade DCIS	3	19%	3	19%	2	13%	8	50%	16	
Total	6	3%	39	17%	133	59%	47	21%	225	
Subsequent invasive grade										
High-grade DCIS	17	8%	83	39%	79	37%	36	17%	215	0.08
Intermediate-grade DCIS	16	11%	78	53%	35	24%	18	12%	147	
Low-grade DCIS	6	12%	28	57%	6	12%	9	18%	49	
DCIS unknown grade	0	0%	1	50%	1	50%	0	0%	2	
Total	39	9%	190	46%	121	29%	63	15%	413	

In view of the present UK^16^ (and international^17^) guidance identifying that a 2-mm width of uninvolved tissue is desirable for DCIS excision, we analysed ipsilateral recurrences (DCIS and invasive) against margin status (<2 mm or ≥2 mm) following BCS. There was a statistically significant lower rate of recurrence for lesions with ≥2 mm clear margin (*p* = 0.003). Further analysis showed that the protective effect of the wider margin width was predominantly on ipsilateral invasive recurrence, both for patients who received or did not receive radiotherapy (*p* = 0.03 and 0.04, respectively) and not on subsequent DCIS recurrence. On analysing margin status and grade of DCIS with ipsilateral recurrence, margin width had a significant impact on recurrence for high-grade DCIS but not of intermediate/low-grade disease. Patients with high-grade DCIS with <2 mm margin had a 6% recurrence rate (for both in situ and invasive carcinoma), whereas those with lesions with ≥2 mm margin had DCIS and invasive recurrence rates of 4 and 5%, respectively. This difference was statistically significant (*p* = 0.02).

The architecture of the DCIS, the presence of comedo necrosis and the presence of additional atypia or of microinvasion were not significantly associated with ipsilateral or contralateral recurrence.

The rate of distant metastasis as first recurrence, without evidence of primary invasive disease, was low (28 patients); of these, 12 (43%) occurred in the first 5 years. The median time to presentation with distant metastasis was 47.5 months.

### Time to subsequent events

**Fig. 1:**
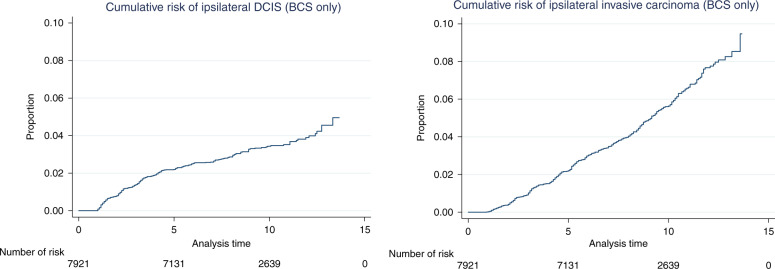
Risk of development of subsequent ipsilateral DCIS (left) and invasive carcinoma (right) in women receiving breast-conserving surgery (BCS) by year. The steady increase in risk of invasive disease continues after 10 years of follow-up.

Kaplan–Meier survival analysis showed distinct patterns for risk of ipsilateral events. While the risk of ipsilateral DCIS tailed off after 5 years, the risk of subsequent invasion showed a consistent year on year increase over 10 years (Fig. 1).

### Effect of adjuvant radiotherapy

**Fig. 2:**
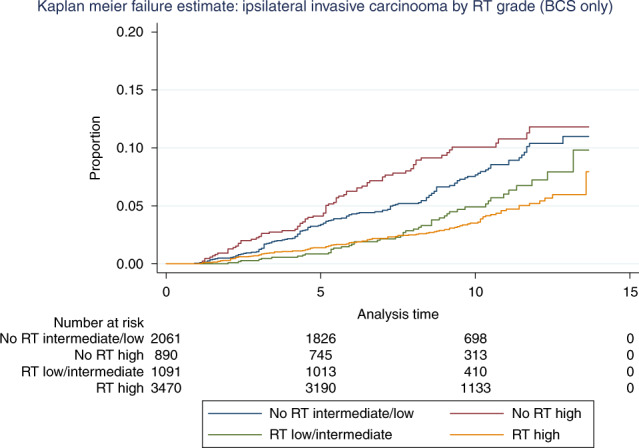
The effect of radiotherapy (RT) on ipsilateral subsequent invasive carcinoma in patients treated with breast-conserving surgery (BCS). Radiotherapy markedly reduced the risk of subsequent ipsilateral invasive carcinoma in patients with primary high-grade DCIS and, to a lesser extent, in the low/intermediate-grade category.

A protective effect of radiotherapy on recurrence was seen, both for in situ and invasive ipsilateral carcinoma. Excluding patients without information on radiotherapy status, recurrence rate for high-grade DCIS treated with adjuvant radiotherapy was 3.2% compared to 6.9% without radiotherapy. The recurrent DCIS rates after low/intermediate-grade DCIS diagnosis with and without radiotherapy were 2.3 and 3.0%, respectively. Radiotherapy reduced subsequent invasive carcinoma rates from 8.9 to 3.7% for high-grade DCIS and from 15 to 7.7% for low/intermediate-grade DCIS. Patients with high-grade DCIS who did not receive radiotherapy had the highest rate of subsequent ipsilateral invasion, whereas those with high-grade DCIS lesions who received adjuvant radiotherapy had the lowest rate (Fig. 2). However, when there was such an invasive disease, it was at a median 56 months following radiotherapy for high-grade DCIS. This protective effect of radiotherapy persisted after exclusion of patients who received endocrine therapy (Fig. 1c).

## Discussion

This analysis provides an overview of a large, prospective cohort of well-characterised DCIS diagnosed in women of screening age within the NHS BSP and is anticipated to be representative of screen-detected DCIS in general. At its inception, the UK NHS BSP was available to all women aged 50–64 years but the upper age limit was increased to age 70 years during 2003–2005. The data include real-life pathology parameters supplied by the UK Breast Screening Units that contributed to the Sloane Project and thus provide a unique opportunity to analyse trends and changes in DCIS reporting and the clinical management implications over the course of a decade.

The small but steady increase in DCIS size (from 21.4 to 24.1 mm) over the period was predominantly due to an increase in the diagnosis of lesions measuring ≥30 mm. This has implications for surgical management, as the majority of those patients with larger DCIS underwent mastectomy. Subsequent to this series, oncoplastic surgical procedures are increasingly being introduced for large volume lesions, and conversely, fewer patients may now therefore require mastectomy. The increase in DCIS size identified between 2003 and 2012 may reflect improvements in imaging modalities over this time (including the introduction of digital mammography), together with more extensive sampling of surgical excision specimens by pathologists, as advised in the current UK guidelines.^14^ Pre-operative assessment guidance now includes the recommendation for sampling more than one focus of a mammographically large area of calcification (>30 mm) to assess the extent of disease, which may also contribute to more accurate assessment of DCIS size.^18^

Over the period presented, there has been an increase in the proportion of high-grade DCIS (from 59 to 63%) and a decrease in the diagnosis of low-grade DCIS (from 10 to 6%). While national guidance on grading has continually been updated,^14^ the consistency of grading of DCIS within the UK External Quality Assurance Scheme remains only in the moderate range with a kappa value of 0.55 for high-grade DCIS.^19^ Studies from other countries have shown low consistency in grading but have also highlighted different distribution of DCIS grades to those seen in this UK series. For example, in the nationwide Dutch Pathology Registry, 4952 DCIS reports from 36 laboratories were analysed: 12.5% were reported as low grade (range 6.1–24.4%), 39.5% as intermediate grade (18.2–57.6%), and 48.0% as high-grade DCIS (30.2–72.7%).^20^ The reasons for the variation in grade of DCIS between countries and screening programmes remain unclear, and several international studies of grading reproducibility are underway to determine whether this predominantly reflects differences in pathological assessment.^21^ An alternative two-tier grading system (high vs non-high grade) has been proposed;^22^ however, one recent international study of 149 DCIS cases assessed by 39 breast pathologists using such a dichotomous grading system still showed only moderate agreement (kappa = 0.422).^23^ As several worldwide trials are presently being undertaken of active surveillance vs surgical intervention for low-risk DCIS,^12^ including the LORIS,^9^ LORD^10^ and the COMET trials^11^, the reproducible grading of DCIS is increasingly important for clinical management and methods for improving reproducibility require further exploration.

In this real-world national prospective data set, only 3% (233) of patients with BCS had margin clearance described as 0 mm (‘involved’), with a further 3% having disease <1 mm from the margin. The UK recommendations of minimum margin clearance have evolved over the years. Both the latest UK NICE^16^ and US guidelines^17^ presently recommend 2 mm clearance for relevant (circumferential) margins of pure DCIS. This cut-off was being achieved in at least 80% of the Sloane cohort.

While classical lobular neoplasia and ADH were both most frequently seen in association with high-grade DCIS (53% of the total cases with lobular neoplasia as the only other atypical lesion and 39% of the total cases with ADH alone), this is a reflection of high-grade DCIS being the most frequently reported grade. As part of the low nuclear grade neoplasia family,^24^ it is not surprising that additional atypias were proportionately more frequently associated with intermediate- or low-grade DCIS (12% of total of lobular neoplasia and 21% of ADH) compared with high-grade DCIS (4% of lobular neoplasia and 3% of ADH). In a further 111 patients, both ADH and lobular in situ neoplasia were present along with the DCIS, again more commonly seen with low-grade DCIS (4% of low-grade DCIS cases) than in intermediate- (1%) or high-grade disease (<1%).

The reporting of ADH in association with low-grade DCIS is controversial. ADH is a microfocal, low-grade atypical lesion with complete involvement of less than two membrane-bound spaces or <2 mm in extent^15^ but is recognised to show similar genetic and biomarker profiles to low-grade DCIS. Although cytologically at least part of the spaces will have the features of low-grade DCIS, this is insufficient in extent for diagnosis of established DCIS. However, if such partial duct involvement is seen adjacent to a larger, more established lesion, many would regard the entire process as low-grade DCIS and not report the two lesions separately. It is not, however, clear whether in the 611 cases, where both DCIS and ADH were recorded in the present database, the processes were in continuity or separate, synchronous lesions. The co-existence of differing grades of DCIS in an individual patient has been described, and some even believe that ‘poorly differentiated’ DCIS may evolve from ‘well-differentiated’ DCIS by randomly acquiring genetic defects,^25^ although this is not widely accepted. Other series have shown greater consistency of grade (85%) in individual patients than uniformity of architecture.^26^ In the Sloane Project, as per UK guidelines, the highest reported histological grade was recorded but the co-existence of classical lobular neoplasia and ADH with high-grade DCIS is noteworthy and merits further investigation of genomic changes present in these cases.

Microinvasion, defined by the UK NHS BSP surgical reporting guidelines as one or more invasive foci measuring <1 mm, is a rare lesion that is recognised to be most commonly seen in the context of high-grade DCIS.^14^ In the present data, it was also identified in association with intermediate- or low-grade DCIS in 4% of cases; the recent WHO breast guidelines also highlight this infrequent existence in non-high-grade lesions.^15^ The frequency of microinvasion in the present series decreased significantly from 2003/2004 to 2011/2012, which may reflect adherence to more stringent criteria for diagnosing microinvasion and the updated NHS BSP pathology guidelines published in 2005.^27^ However, within the Sloane cohort there was a wide variation in the rate of reporting of microinvasive carcinoma between centres (0–29%). This may reflect bias in submission of cases to Sloane from individual centres, but the incidence of microinvasion in the literature also varies from as low as 0.68%^28^ to as high as 8.3%.^29^ The high frequency in some Units submitting data to Sloane also highlights questions about the reproducibility of this diagnosis. The definition of microinvasion as necessarily in the non-specialised stroma was included in previous UK guidelines^27^ but has been excluded from more recent updated version.^14^ Various definitions have been used (for a review, see Bianchi and Vezzosi^30^) and the latest WHO book does not include this criterion.^15^

Another histopathological feature that lacks consistency in its definition and reporting is comedo necrosis; criteria used range from any central necrosis to expansive necrosis. The definition of comedo necrosis has been the subject of recent debate, particularly the minimum amount of central necrosis required to qualify as ‘comedo necrosis’. One recent survey of 35 experienced breast pathologists from the USA showed that no single cut-off was agreed by more than a third of participants;^31^ the minimum threshold of cross sectional necrosis required ranged from 10% (by 4 pathologists) to 70% (by 1 pathologist). Currently, there is no clinical evidence for recommending one threshold over any other and the descriptive term ‘central necrosis’ with comment regarding number, or proportion, of ducts involved may be more appropriate in describing this histological feature, as recently recommended by the International Collaboration on Cancer Reporting DCIS data set.^32^

**Fig. 3:**
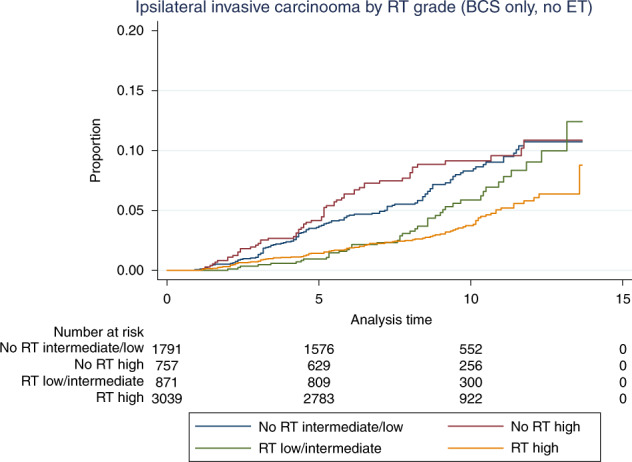
Ipsilateral invasive recurrence by receipt of radiotherapy (RT) and grade of DCIS in women who underwent breast-conserving surgery. Without RT or endocrine therapy (ET), invasive carcinoma rates are higher in the high grade than in those with low/intermediate-grade DCIS up to 10 years.

Follow-up data of the patients from England is the largest component of this prospective cohort. The screening programme is the same throughout the United Kingdom, and clinical management guidelines are essentially similar. Therefore, we believe the data are generalisable to the UK population, as well as potentially further afield. In the cohort with extended follow-up (9191 patients), it is intriguing that a higher rate of subsequent in situ and invasive carcinoma is noted following a primary diagnosis of either low- or intermediate-grade DCIS compared with high-grade disease. This is contrary to data from series of untreated DCIS, where a higher and more rapid rate of progression to invasive disease is seen for high-grade DCIS left in situ, i.e. not excised.^33^ However, when analysis is limited to the majority of patients who have undergone BCS (7934, 86% of patients) the ipsilateral recurrence rates are 9.2, 9.7 and 9.8%, respectively, for high, intermediate- and low-grade DCIS (with invasive recurrence frequencies of 4.9, 6.7 and 6.7%, respectively). More patients with high-grade DCIS undergo mastectomy or when receiving BCS are in receipt of radiotherapy,^34^ which is known to reduce ipsilateral recurrence.^35^ Indeed, an increased sensitivity of high-grade DCIS to radiotherapy may be deduced from the present data (Figs. 2 and 3), which would contribute to the lower recurrence rate seen with high-grade DCIS after BCS. The differences may also be influenced by the ability to determine DCIS size and especially completeness of excision more accurately in high-grade DCIS, due to a greater proportion of ducts associated with microcalcification when compared to low-grade DCIS in which a greater proportion of the area is non-calcific.^36^ Theoretically, for this reason, a greater proportion of low-grade DCIS may therefore be incompletely excised, albeit unrecognised on radiological (specimen X-ray) and pathological examination.

In this cohort, the grade of contralateral DCIS or invasive carcinoma did not necessarily mirror the grade of the primary DCIS, consistent with contralateral breast cancer representing independent, new disease, rather than recurrence. Recent molecular analysis of paired small cohorts of DCIS and invasive carcinomas within the Sloane cohort supports this hypothesis.^37^ While grade of subsequent DCIS was associated with grade of primary DCIS, the grade of subsequent ipsilateral invasive cancer showed a non-significant association with the original DCIS grade (Table 5). Data on the pleomorphism component of the invasive histological grade is not collected in the Sloane database, but it is likely that the DCIS cytonuclear grade and pleomorphism score may show a greater correlation than overall histological invasive grade.

A novel finding is this study is the tapering of risk of subsequent ipsilateral DCIS over time, while the risk of invasive carcinoma in the same breast continued. We believe that this finding is important and relevant for the need to undertake long-term follow-up strategies with DCIS and for informed patient decisions on management. A recent epidemiological study has also identified that invasive carcinoma continues to develop up to 20 years following the diagnosis and treatment of DCIS.^38^ More of the subsequent events in our series occurred as invasive carcinoma. This current data therefore differs from earlier reports (e.g. EBCTCG, EORTC) that recorded that approximately half of the ipsilateral breast recurrences after a diagnosis of pure DCIS were as invasive disease and half as DCIS, with a 50% reduction in either form of recurrence following radiotherapy.^35^ This was based on trials such as NSABP–B17 and EORTC 10853 that included patients treated between 1985 and 1999. Since then, considerable advances have been made in refining the radiological and histological diagnosis, tissue sampling, classification of DCIS and margin assessment. There has also, however, been progress in surgical treatment and in revisiting the significance of margin status, as well as selection of patients for radiotherapy. We believe that the present data are reflective of current practice, both histopathological and clinical, and that this change in the nature of recurrence is likely to be multifactorial.

The main strengths of this study include the prospective collection of a wide range of pathological features of large numbers of screen-detected DCIS over time. The link to multiple national information systems, as well as the provision of recurrence data from the individual NHS BSP centres, uniquely allows correlation of the histological features of primary DCIS with those of subsequent events. However, one caveat in interpreting these recurrence data is the length of follow-up in these analyses, given that, even with approaching 10 years of follow-up, further events are certain to occur in this patient population over subsequent decades.

In conclusion, we provide a comprehensive overview of the pathology features of screen-detected DCIS, including information on recurrence of disease that should inform strategies for DCIS management, patient counselling and follow-up. The protective effect of radiotherapy is confirmed on all DCIS grades but is particularly important in high-grade disease. Issues related to the reproducibility of some pathological features including comedo necrosis and microinvasion should be noted; further work to define the most clinically relevant cut-offs for some features and subsequent updates to international guidelines are required. While grade of DCIS has previously been of limited clinical application, the ongoing trials of surveillance of low-risk DCIS have highlighted the importance of reproducibility of this key pathological feature.

## Data Availability

Data are held by Public Health England. Access to the Sloane Project data from external parties is governed by consultation with the Sloane Project Steering Group and application to Public Health England’s breast screening research advisory committee (RAC) and Public Health England’s office for data release (ODR). Data will subsequently only be released by Public Health England to researchers under approval and in an anonymised or depersonalised format, with a data sharing contract in place.
